# Diagnostic performance of integrated whole-body ^18^F-FDG PET/MRI for detecting bone marrow involvement in indolent lymphoma: Comparison with ^18^F-FDG PET or MRI alone

**DOI:** 10.3389/fonc.2023.1136687

**Published:** 2023-03-13

**Authors:** Xuetao Chen, Tingting Yuan, Maomao Wei, Boqi Yu, Nina Zhou, Hua Zhu, Zhi Yang, Xuejuan Wang

**Affiliations:** Key Laboratory of Carcinogenesis and Translational Research (Ministry of Education/Beijing), National Medical Products Administration (NMPA) Key Laboratory for Research and Evaluation of Radiopharmaceuticals, Department of Nuclear Medicine, Peking University Cancer Hospital and Institute, Beijing, China

**Keywords:** integrated whole-body 18 F-FDG PET/MRI, bone marrow involvement, indolent lymphoma, PET, MRI, bone marrow biopsy

## Abstract

**Purpose:**

To investigate the diagnostic performance of integrated whole-body ^18^F-FDG PET/MRI for detecting bone marrow involvement (BMI) in indolent lymphoma compared with ^18^F-FDG PET or MRI alone.

**Methods:**

Patients with treatment-naive indolent lymphoma who underwent integrated whole-body ^18^F-FDG PET/MRI and bone marrow biopsy (BMB) were prospectively enrolled. Agreement between PET, MRI, PET/MRI, BMB, and the reference standard was assessed using kappa statistics. The sensitivity, specificity, accuracy, positive predictive value (PPV), and negative predictive value (NPV) of each method were calculated. A receiver operating characteristic (ROC) curve was used to determine the area under the curve (AUC). AUCs of PET, MRI, PET/MRI, and BMB were compared using the DeLong test.

**Results:**

Fifty-five patients (24 males and 31 females; mean age: 51.1 ± 10.1 years) were included in this study. Of these 55 patients, 19 (34.5%) had BMI. Two patients were upstaged as extra bone marrow lesions were detected *via* PET/MRI. 97.1% (33/34) of participants were confirmed as BMB-negative in the PET-/MRI-group. PET/MRI (parallel test) and BMB showed excellent agreement with the reference standard (k = 0.843, 0.918), whereas PET and MRI showed moderate agreement (k = 0.554, 0.577). The sensitivity, specificity, accuracy, PPV, and NPV for identifying BMI in indolent lymphoma were 52.6%, 97.2%, 81.8%, 90.9%, and 79.5%, respectively, for PET; 63.2%, 91.7%, 81.8%, 80.0%, and 82.5%, respectively, for MRI; 89.5%, 100%, 96.4%, 100%, and 94.7%, respectively, for BMB; and 94.7%, 91.7%, 92.7%, 85.7%, and 97.1%, respectively, for PET/MRI (parallel test). According to ROC analysis, the AUCs of PET, MRI, BMB, and PET/MRI (parallel test) for detecting BMI in indolent lymphomas were 0.749, 0.774, 0.947, and 0.932, respectively. The DeLong test showed significant differences between the AUCs of PET/MRI (parallel test) and those of PET (P = 0.003) and MRI (P = 0.004). Regarding histologic subtypes, the diagnostic performance of PET/MRI for detecting BMI in small lymphocytic lymphoma was lower than that in follicular lymphoma, which was in turn lower than that in marginal zone lymphoma.

**Conclusion:**

Integrated whole-body ^18^F-FDG PET/MRI showed excellent sensitivity and accuracy for detecting BMI in indolent lymphoma compared with ^18^F-FDG PET or MRI alone, demonstrating that ^18^F-FDG PET/MRI is an optimal method and a reliable alternative to BMB.

**Trial registration:**

ClinicalTrials.gov (NCT05004961 and NCT05390632)

## 1 Introduction

Indolent lymphoma, a set of malignancies of B-lymphocytes, comprises 35%–45% of non-Hodgkin’s lymphomas and is associated with a low proliferative rate that logically explains the typical long median survival of these patients (1). Bone marrow involvement (BMI) implies the highest disease stage (stage IV) according to the Ann Arbor classification (2), which is considered an adverse prognostic factor (3). Guidelines for indolent lymphomas still require routine staging BMB, although the procedures are inconsistently performed in clinical practice. The National Comprehensive Cancer Network guidelines specifically state that the procedures may be discontinued for those who are being followed without treatment. A recent study indicated that BMB should be removed from the diagnostic guidelines for FL (follicular lymphoma), except in cases wherein it may change management, such as the confirmation of limited-stage disease and assessment of cytopenia (4). Meantime, BMB is an invasive examination that causes pain, anxiety, and other adverse events; furthermore, it may miss patchy infiltration due to its restriction to the iliac crest/site of biopsy (5).

The multimodality imaging may provide more information and help clinicians to evaluate bone marrow status. ^18^F-fluorodeoxyglucose (FDG) positron emission tomography (PET), which enables the visualization of the entire marrow, has recently emerged as an established technique for detecting BMI in many aggressive lymphoma subtypes, as it is based on increased glucose metabolism (6, 7). However, the clinical utility of ^18^F-FDG PET was limited to distinguish BMI from normal bone marrow in non-FDG-avid lymphoma subtypes. Several studies with large study cohorts reported that the sensitivity of PET in detecting BMI in follicular lymphoma (FL) was approximately 30% (8, 9) owing to low or absence of FDG uptake (10). Because malignant lesions show higher signal intensity than normal tissues in diffusion-weighted imaging (DWI) owing to their high cellularity, which restricts the diffusion of water molecules, whole-body MRI with DWI (MRI-DWI) has been widely adopted to evaluate bone marrow primary and metastatic diseases without any additional radiation. However, few studies have reported that MRI detects BMI in indolent lymphoma, and whether or not MRI aids in the assessment of BMI in indolent lymphoma remains debatable (11–13).

Integrated whole-body PET/MRI is a reliable alternative to conventional imaging methods in the staging of patients with lymphoma, which enables the simultaneous acquisition of PET and MRI data, thereby combining the advantages of the metabolic information of PET with the high contrast resolution of MRI for soft tissue and bone marrow (14, 15). Thus, this prospective study aimed to explore the diagnostic performance of integrated whole-body ^18^F-FDG PET/MRI for detecting BMI in indolent lymphoma.

## 2 Materials and methods

### 2.1 Patient enrollment

This single-center prospective study was approved by the Institutional Review Board of the Peking University Cancer Hospital and Institute (No. 2018KT110-GZ01) and was registered with ClinicalTrials.gov (NCT05004961 and NCT05390632). Patient enrollment was performed consecutively from September 2020 to April 2021. Written informed consent was obtained from all patients. The inclusion criteria were as follows: (a) newly diagnosed indolent lymphoma, (b) age 18–75 years, (c) expected survival duration of ≥12 weeks, and (d) availability of complete medical history and clinicopathological data. The exclusion criteria were as follows: (a) unwillingness to provide informed consent, (b) pregnancy or lactation, (c) a history of secondary malignancies, (d) severe liver or kidney dysfunction, (e) intolerance to long-term supine positioning, and (f) other contraindications for MRI (claustrophobia, metal implants, electronic devices, etc.).

### 2.2 ^18^F-FDG PET/MRI imaging

^18^F-FDG was administered intravenously at a dose of 3.7–4.4 MBq/kg after patients fasted for at least 6 h, with blood glucose levels <10 mmol/L (range, 4.2–8.9 mmol/L). PET and MRI scans were acquired simultaneously, which ranged from the base of the skull to the upper thigh, using an integrated PET/MRI system (uPMR 790, United Imaging Healthcare, Shanghai, China) equipped with a 12-channel body coil, combining a time-of-flight PET scanner and 3.0-T MRI. The mean time interval between the injection and image acquisition was 60 ± 15 min. The acquisition time of PET/MRI was 50–60 min. The spine imaging protocol included T1-weighted imaging (T1WI), T2-weighted imaging (T2WI; axial and sagittal acquisition), and DWI with b-values of 50 and 800 s/mm^2^.

### 2.3 BMB

The mean time interval between BMB and ^18^F-FDG PET/MRI examinations was 2 weeks. Unilateral BMB was performed at the level of the posterior iliac crest along with immunohistochemistry and flow cytometry, and BMI for lymphoma was interpreted by two pathologists at our hospital who were blinded to the PET/MRI results.

### 2.4 Image analysis

**Figure 1:**
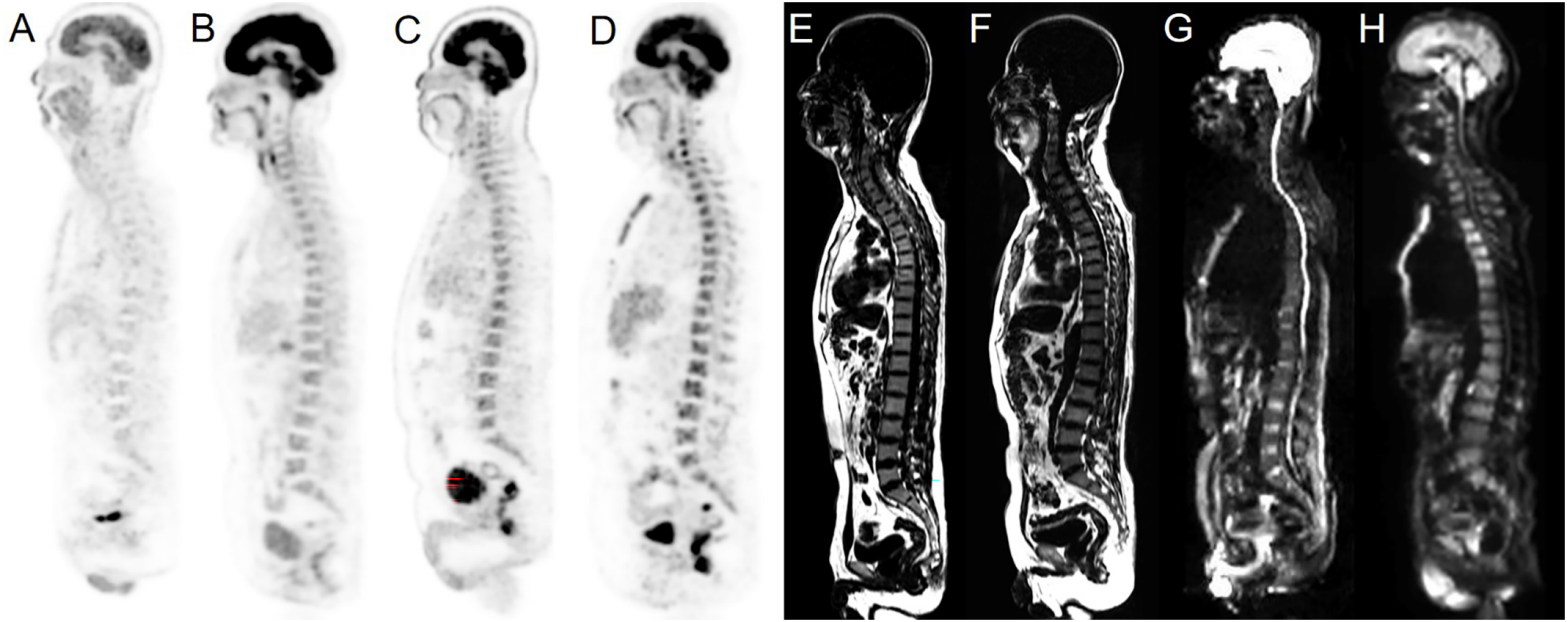
Examples of normal BMs and BMI in our cohort. Sagittal PET images derived from integrated PET/MRI **(A–D)**, T1WI and sagittal DWI images derived from integrated PET/MRI **(E–H)**. Normal **(A)** and homogeneously increased ^18^F-FDG uptake above the liver parenchyma **(B)** were considered PET-negative. Heterogeneously increased **(C)** and unifocal/multifocal FDG uptake above the liver parenchyma uptake **(D)** were considered PET-positive. Normal signals of BM on the sagittal T1WI image were considered MRI-negative **(E)**. T1WI positivity was defined as decreased signal intensity compared to the surrounding muscles **(F)**. Normal signals of BM on the sagittal DWI image were considered MRI-negative **(G)**. DWI positivity was defined as increased signal intensity compared to the surrounding muscles and intervertebral discs **(H)**.

The ^18^F-FDG PET/MRI scans were visually evaluated by three blinded experienced nuclear physicians and a radiologist in consensus (with 8, 10, 24, and 15 years of experience, respectively). Images with heterogeneously increased or unifocal/multifocal FDG uptake above liver parenchyma were classified as PET-positive (16), while those with homogeneously increased or normal FDG uptake were considered PET-negative. Images were considered MRI-positive if there were areas characterized by the decreased signal intensity on T1WI and higher signal intensity on T2WI and DWI than that of the surrounding muscles and intervertebral discs (**Figure 1**). PET/MRI was classified into two patterns: parallel test and serial test. PET/MRI (parallel test) was considered positive when BMI was detected *via* either PET or MRI derived from integrated PET/MRI, while PET/MRI (serial test) was considered positive when BMI was detected *via* both PET and MRI. The parallel and serial tests were used to determine the optimal diagnostic performance of integrated ^18^F-FDG PET/MRI for detecting BMI.

### 2.5 Reference standard

The reference standard BMI was determined through BMB or/and follow-up multimodality imaging techniques (i.e., CT, MRI, ^18^F-FDG PET/CT, and/or ^18^F-FDG PET/MRI) (17).

### 2.6 Statistical analysis

Continuous variables are presented as means ± standard deviations. SPSS (version 23.0, IBM Corp.) and MedCalc (version 20.0.26, MedCalc Software Ltd.) were used for statistical analyses. Agreement between the abovementioned techniques and the reference standard was assessed using kappa statistics. Kappa values were indicative of poor (k < 0.2), fair (k = 0.21–0.40), moderate (k = 0.41–0.60), good (k = 0.61–0.80), and excellent (k > 0.8) agreement. The sensitivity, specificity, accuracy, positive predictive value (PPV), and negative predictive value (NPV) of each method were calculated and analyzed. Diagnostic efficiency was assessed using receiver operating characteristic curves (ROCs), which were used to determine areas under the curve (AUCs). AUCs of PET, MRI, PET/MRI, and BMB were compared using the DeLong test. A *p*-value of <0.05 was considered statistically significant.

## 3 Results

### 3.1 Patient characteristics

Fifty-five patients (24 males and 31 females; mean age: 51.1 ± 10.1 years [range: 29–77 years]) were included in this study. FL was the most common indolent lymphoma subtype (detected in 83.6% of patients). The clinical characteristics of the 55 study patients are presented in **Table 1**. A total of 19 (34.5%) patients were considered as having BMI per the reference standard. 12.7% of patients were classified as early-stage, while 87.3% were identified as advanced-stage.

**Table 1:** Clinical characteristics of 55 patients with indolent lymphoma.

Parameters	No. of Patients (%)
Sex	
Male	24 (44.6%)
Female	31 (56.4%)
Age	
>60 years	10 (18.2%)
≤60 years	45 (81.8%)
BMI	
Positive	19 (34.5%)
Negative	36 (65.5%)
Stage	
I–II	7 (12.7%)
III–VI	48 (87.3%)
Histological subtype	
Follicular lymphoma	46 (83.6%)
Marginal zone lymphoma	6 (10.9%)
Small lymphocytic lymphoma	3 (5.5%)

### 3.2 Visual analysis

According to the standard reference, two patients (3.6%) were upstaged as extra bone marrow lesions were detected *via* PET/MRI, which were proven by follow-up imaging techniques. In 17 patients (30.9%) with stage IV disease, the stage did not change despite the detection of BMI *via* PET/MRI. Other patients remained at the same stage.

PET features with normal, homogeneous increased, heterogeneously increased, and uni-/multifocal FDG uptake were identified in 27 (49.1%), 10 (18.2%), 7 (12.7%), and 2 (3.6%) patients with FL, respectively, and in 3 (5.5%), 1 (1.8%), 1 (1.8%), and 1 (1.8%) patients with marginal zone lymphoma (MZL), respectively. Bone marrow FDG uptake in small lymphocytic lymphoma (SLL; 5.5%) was lower than or similar to liver FDG uptake. 37.5% of patients with FL and 50.0% of those with MZL showed heterogeneous FDG uptake in the bone marrow on PET, whereas all patients with SLL showed homogeneous uptake.

According to imaging profiles, PET/MRI was divided into four groups (PET+/MRI+, PET+/MRI−, PET−/MRI+, and PET−/MRI−), and each group had 5 (9.1%), 6 (10.9%), 10 (18.2%), and 34 (61.8%) patients, respectively. In the PET+/MRI− group, all patients had the FL subtype. Two SLL and 8 FL patients presented PET−/MRI+ profiles. Four out of five patients presented PET+/MRI+ features and were confirmed to have BMI through BMB. In the PET+/MRI− and PET−/MRI+ groups, 83.3% (5/6) and 70% (7/10) of patients, were confirmed to be BMB-positive, respectively. Most of the patients (33/34, 97.1%) in the PET−/MRI− group were confirmed to be BMB-negative.

Agreements between PET, MRI and BMB with the reference standard were 81.8% (45/55, 10 positives and 35 negatives), 81.8% (45/55, 12 positives and 33 negatives), and 96.4% (53/55, 17 positives and 36 negatives) in patients with indolent lymphoma, respectively. Moreover, PET/MRI (parallel test) and PET/MRI (serial test) was consistent with the reference standard in 92.7% (51/55, 18 positives and 33 negatives) and 70.9% (39/55, 4 positives and 35 negatives) of patients, respectively (**Table 2**). Concordance analyses revealed excellent agreement between PET/MRI (parallel test) and BMB with the reference standard in this cohort (k = 0.843, 0.918); PET and MRI showed moderate agreement with the reference standard (k = 0.554, 0.577); PET/MRI (serial test) showed the poorest agreement with the reference standard (k = 0.221). The detection rate of PET/MRI (parallel test) was significantly higher than those of PET, MRI, and PET/MRI (serial test; P = 0.001, 0.031, 0.000, respectively). No significant difference was found between PET/MRI (parallel test) and BMB in detecting BMI (P = 0.219).

**Table 2:** Comparison of PET, MRI, PET/MRI, and BMB results with the reference standard for detecting bone marrow involvement.

Diagnostic Modality		Reference standard		
		(+)	(−)	Total
PET	(+)	10	1	11
	(−)	9	35	44
MRI	(+)	12	3	15
	(−)	7	33	40
PET/MRI (parallel test)	(+)	18	3	21
	(−)	1	33	34
PET/MRI (serial test)	(+)	4	1	5
	(−)	15	35	50
BMB	(+)	17	0	17
	(−)	2	36	38
Total		19	36	55

### 3.3 Diagnostic efficiency

**Figure 2:**
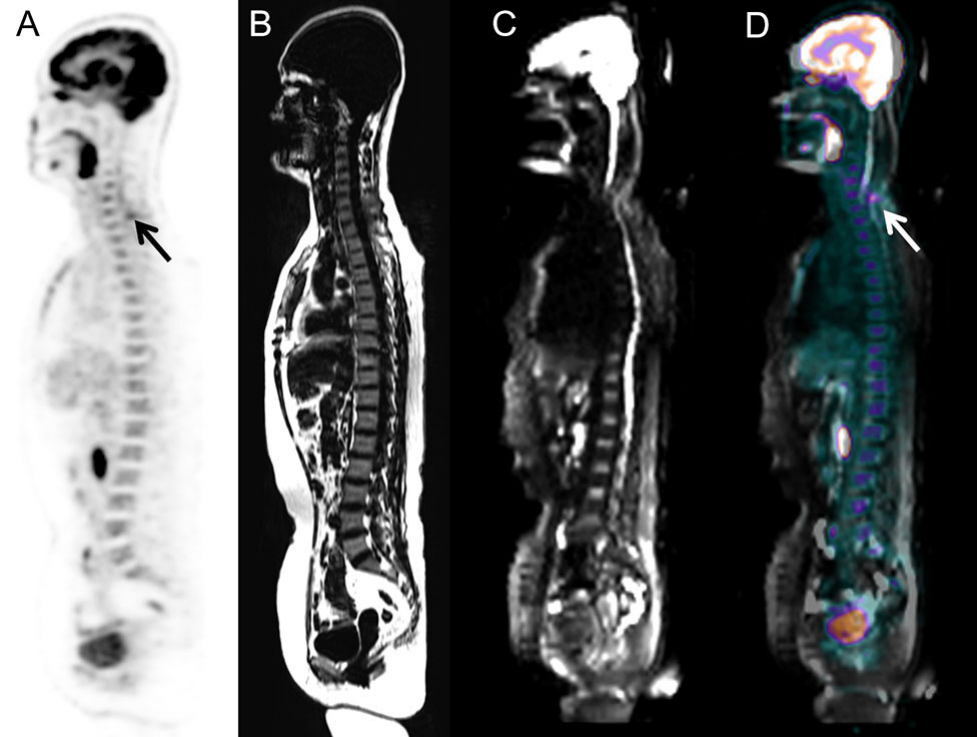
Images of a 58-year-old woman with low-grade follicular lymphoma. BMI was detected via PET/MRI according to the unifocal intense uptake of FDG on the PET image, while MRI did not show any intense signal because of artifacts. The presence of BMI was also confirmed through BMB. **(A)** PET image derived from integrated PET/MRI. **(B)** TIWI image derived from integrated PET/MRI. **(C)** DWI image derived from integrated PET/MRI. **(D)** Fused whole-body integrated PET/MRI.

**Figure 3:**
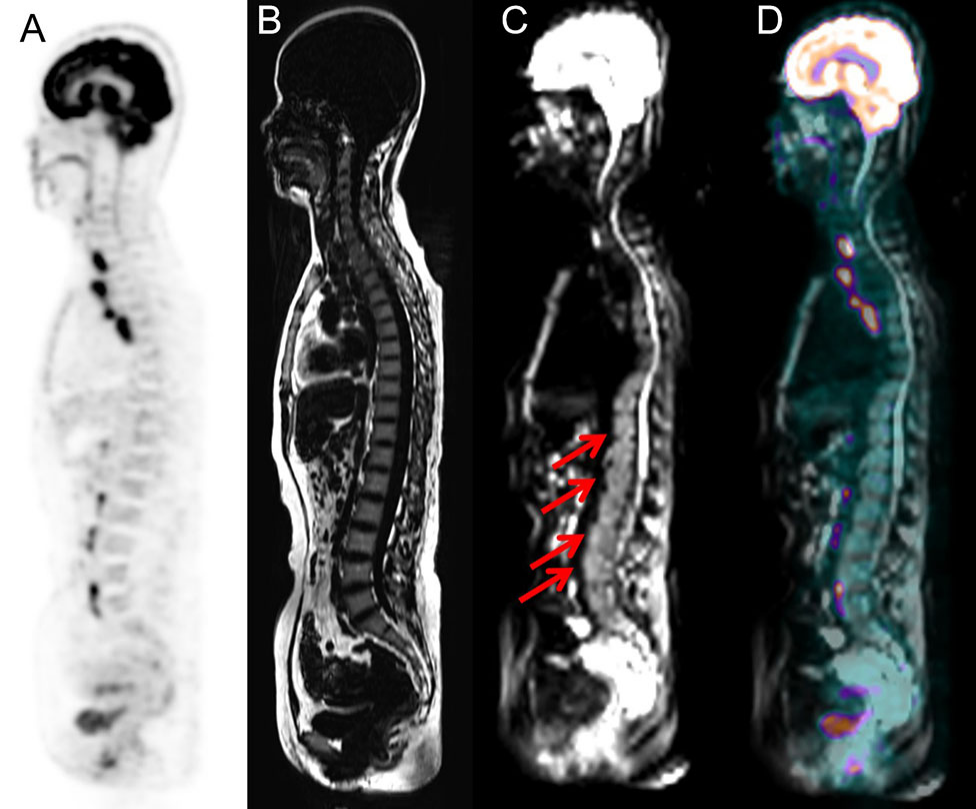
Images of a 51-year-old woman with small lymphocytic lymphoma. BMI was detected via PET/MRI due to the presence of intense signal on the MRI (arrows), while PET did not show heterogeneously high FDG uptake. The presence of BMI was confirmed by routine staging BMB, which was performed at the level of the posterior iliac crest. **(A)** PET image derived from PET/MRI. **(B)** TIWI image derived from integrated PET/MRI. **(C)** DWI image derived from integrated PET/MRI. **(D)** Fused whole-body integrated PET/MRI.

Heterogeneous FDG-avid foci (maximum standardized uptake value [SUV_max]_, range 2.2–5.6) in the cervical, thoracic, and/or lumbar vertebrae were detected in six patients with normal MRI-DWI signal intensity (**Figure 2**), and all patients were confirmed to have BMI through BMB (5 patients) and follow-up imaging techniques (1 patient). Ten patients with negative ^18^F-FDG PET findings showed increased spine signal intensity on MRI-DWI, while only 80% (8/10) patients were confirmed to have BMI (7 *via* BMB and 1 by follow-up; **Figure 3**). **Table 3** shows the diagnostic performance of each modality for detecting BMI. The sensitivity, specificity, accuracy, PPV, and NPV for identifying BMI in indolent lymphoma were 52.6%, 97.2%, 81.8%, 90.9%, and 79.5%, respectively, for PET; 63.2%, 91.7%, 81.8%, 80.0%, and 82.5%, respectively, for MRI; 89.5%, 100%, 96.4%, 100%, and 94.7%, respectively, for BMB; 94.7%, 91.7%, 92.7%, 85.7%, and 97.1%, respectively, for PET/MRI (parallel test); and 21.1%, 97.2%, 70.9%, 80.0%, and 70.0%, respectively, for PET/MRI (serial test).

**Table 3:** Diagnostic efficiency of PET, MRI, PET/MRI, and BMB for detecting bone marrow involvement.

Diagnostic Modality	SE (%)	SP (%)	ACC (%)	PPV (%)	NPV (%)	AUC
PET	52.6	97.2	81.8	90.9	79.5	0.749
MRI	63.2	91.7	81.8	80.0	82.5	0.774
PET/MRI (parallel test)	94.7	91.7	92.7	85.7	97.1	0.932
PET/MRI (serial test)	21.1	97.2	70.9	80.0	70.0	0.591
BMB	89.5	100	96.4	100	94.7	0.947

SE, sensitivity; SP, specificity; ACC, accuracy; PPV, positive predictive value; NPV, negative predictive value; AUC, area under the curve.

**Figure 4:**
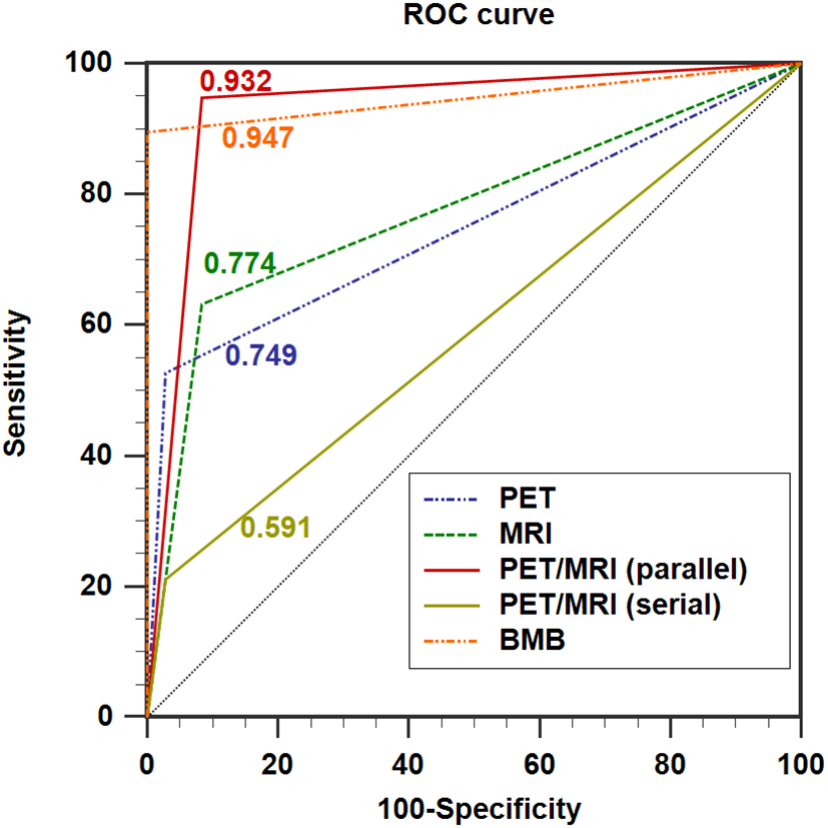
ROC curves showing the diagnostic performance of ^18^F-FDG PET/MRI compared with PET and MRI in detecting BMI in indolent lymphoma. AUC for PET = 0.749 (0.614; 0.856), AUC for MRI = 0.774 (0.641; 0.876), AUC for PET/MRI (parallel test) = 0.932 (0.830; 0.982), AUC for PET/MRI (serial test) = 0.591 (0.451; 0.722), and AUC for BMB = 0.947 (0.851; 0.989). AUC: area under the curve.

In the ROC analysis, the AUCs of PET, MRI, and BMB were 0.749, 0.774, and 0.947, respectively (**Figure 4**); the efficiency of diagnosing BMI was significantly higher in PET/MRI (parallel test) than in PET/MRI (serial test) (AUC: 0.932 *vs*. 0.591). The DeLong test showed a significant difference between the AUCs of PET/MRI (parallel test) and those of PET (P = 0.003) and MRI (P = 0.004; **Table 4**), except BMB (P = 0.768), while the AUC of PET/MRI (serial test) was significantly lower than those of PET (P = 0.004), MRI (P = 0.003), PET/MRI (parallel test; P < 0.001), and BMB (P < 0.001).

**Table 4:** Comparison of AUCs of PET, MRI, PET/MRI, and BMB for detecting bone marrow involvement.

Examination	PET	MRI	PET/MRI (parallel test)	PET/MRI (serial test)
MRI	0.808	N/A		
PET/MRI (parallel test)	0.003	0.004	N/A	
PET/MRI (serial test)	0.004	0.003	< 0.001	N/A
BMB	0.005	0.012	0.768	< 0.001

The sensitivity, specificity, accuracy, PPV, and NPV for identifying BMI in FL were 50.0%, 96.7%, 80.4%, 88.9%, and 78.4%, respectively, for PET; 56.3%, 93.3%, 80.4%, 81.8%, and 80.0%, respectively, for MRI; 87.5%, 100%, 95.7%, 100%, and 93.8%, respectively, for BMB; 93.8%, 93.3%, 93.5%, 88.2%, and 96.6%, respectively, for PET/MRI (parallel test); and 12.5%, 96.7%, 67.4%, 66.7%, and 67.4%, respectively, for PET/MRI (serial test). The AUCs of PET, MRI, BMB, PET/MRI (parallel test), and PET/MRI (serial test) were 0.733, 0.748, 0.938, 0.935, and 0.546, respectively. The sensitivity, specificity, accuracy, PPV, and NPV of each modality were 100% in 6 patients with MZL. All three patients with SLL possessed normal PET profiles, and BMI was detected in only 1 patient (33.3%) using BMB, MRI, and PET/MRI (parallel test).

## 4 Discussion

To the best of our knowledge, this prospective study is the first investigation of the diagnostic performance of integrated whole-body ^18^F-FDG PET/MRI for detecting BMI in patients with indolent lymphoma, and ^18^F-FDG PET/MRI demonstrated excellent sensitivity and accuracy compared with ^18^F-FDG PET or MRI alone.

Prior studies have noted the diagnostic efficiency of PET and MRI for evaluating BMI in lymphoma (18–21). The pooled sensitivity of PET for detecting BMI for indolent lymphoma was 46% (22), which was attributable to the relatively low metabolic rate and FDG uptake per cell or to the diffuse, low-density marrow involvement by the tumor (23). Kim et al. (21) summarized five studies involving 212 lymphoma patients in a systematic review and found that the pooled sensitivity of MRI for detecting BMI was 78%. However, few studies have focused on the use of MRI for evaluating BMI in indolent lymphoma. Given the poor performance of PET or MRI in evaluating bone marrow status in lymphoma, we hypothesized that integrated whole-body ^18^F-FDG PET/MRI might be the diagnostic method of choice for patients with lymphoma, as it combines the valuable metabolic information provided by PET with the excellent soft tissue contrast provided by MRI and avoids radiation exposure from computed tomography. Thus, this study attempted to investigate whether FDG PET/MRI could be an alternate method for characterizing bone marrow status compared with BMB and other imaging modalities.

Our study demonstrated that the sensitivity of integrated PET/MRI for detecting BMI was 94.7%, which was significantly higher than those of PET (52.6%), MRI (63.2%), and BMB (89.5%). Particularly, double-negative PET/MRI (PET−/MRI−) could detect 97.1% of indolent patients with normal bone marrow status, which makes omitting BMB possible. PET/MRI (parallel test), a combination of metabolic evaluation and DWI, exhibited significantly improved diagnostic performance compared with those of individual modalities (PET and MRI), with better sensitivity, NPV, and accuracy (94.7%, 97.1%, and 92.7% *vs*. 52.6%, 79.5%, and 81.8% *vs*. 63.2%, 82.5%, and 81.8%, respectively) and higher AUC (0.932 *vs*. 0.749 *vs*. 0.774). Therefore, these compelling data of PET/MRI (parallel test) add greatest value for detecting BMI in indolent lymphoma and may avoid the side-effect of BMB or additional imaging examinations. Unfortunately, PET/MRI (serial test) was insufficient for detecting BMI in indolent lymphoma. Furthermore, we preliminarily characterized the diagnostic efficiency of PET/MRI for BMI within indolent lymphoma. We found that the sensitivity of integrated PET/MRI (93.8%) was significantly higher than those of PET (50.0%), MRI (56.3%), and BMB (87.5%) in patients with FL. Moreover, PET/MRI showed a false-negative result in one patient with FL, probably because of low lymphomatous infiltration in bone marrow (≤30% of marrow cellularity) as reported by Albano et al. (24). All modalities exhibited excellent diagnostic efficiency for BMI in patients with MZL. Because of a low number of SLL cases in this study, the diagnostic performance of PET/MRI for BMI in SLL should be evaluated in a large-scale clinical trial.

Generally, diffused FDG uptake on PET is considered negative for BMI or red bone marrow hyperplasia, which might result in a higher false-negative rate and a lower sensitivity of detecting BMI (9, 25). Our data demonstrated that 16.7% (4/24) of patients with normal FDG uptake and 40.0% (4/10) of patients with homogeneously increased FDG uptake on PET imaging were confirmed to have BMI *via* PET/MRI and BMB. This piqued our interest in examining the underlying mechanisms of imaging patterns. Some researchers have reported that the histological bone marrow pattern of BMI can be diffuse, nodular, paratrabecular, interstitial, or intrasinusoidal. For instance, Jahic et al. (26) found that diffuse bone marrow pattern was more common in patients with SLL. Sovani et al. (27) reported that the prominent bone marrow pattern of FL was paratrabecular; however, MZL showed a relatively even distribution between these patterns. The association of histological bone marrow pattern with PET/MRI profiles should be investigated in study with a larger cohort.

Owing to the limitations of this study, such as a heterogeneous patient population and a low number of SLL cases, further studies are necessary. Furthermore, prognostic factors were not evaluated. Therefore, larger prospective clinical studies are needed for further evaluation.

In conclusion, integrated whole-body ^18^F-FDG PET/MRI showed excellent sensitivity and accuracy for detecting BMI in indolent lymphoma compared with PET or MRI alone, demonstrating that ^18^F-FDG PET/MRI is an optimal method and a reliable alternative to BMB.

## Data availability statement

The original contributions presented in the study are included in the article/supplementary material. Further inquiries can be directed to the corresponding authors.

## Ethics statement

The studies involving human participants were reviewed and approved by Institutional Review Board of the Peking University Cancer Hospital and Institute. The patients/participants provided their written informed consent to participate in this study.

## Author contributions

Concept and design: XC, XW. Literature search: XC, XW. Data acquisition: BY. Data analysis: XC, MW, NZ, XW. Follow-up data: XC, TY, MW. Radiopharmaceutical synthesis: HZ. Supervision and project administration: ZY. Supervision and Funding acquisition: XW. Manuscript editing: XC, XW. Manuscript review: XC, TY, MW, BY, NZ, HZ, ZY, XW. All authors contributed to the article and approved the submitted version.
